# ARTP mutagenesis for genome-wide identification of genes important for biofilm regulation in spoilage bacterium *Pseudomonas fluorescens* PF08

**DOI:** 10.1128/aem.00218-25

**Published:** 2025-05-07

**Authors:** Feifei Wang, Shenjia Chen, Jinjing Zhou, Ruiyu Zhu, Jian Chen, Yanbo Wang

**Affiliations:** 1 School of Biological and Chemical Engineering, Zhejiang University of Science and Technology 91616, Hangzhou, Zhejiang, China; 2 Food Safety Key Laboratory of Zhejiang Province, School of Food Science and Biotechnology, Zhejiang Gongshang University 12625, Hangzhou, Zhejiang, China; 3 School of Food and Health, Beijing Technology and Business University 578523, Beijing, Beijing, China; Universita degli Studi di Napoli Federico II, Portici, Italy

**Keywords:** ARTP mutagenesis, *Pseudomonas fluorescens*, biofilm, c-di-GMP, siderophore

## Abstract

*Pseudomonas fluorescens* is a vital food spoilage bacterium and commonly spoils foods in the form of biofilms. Yet its biofilm regulation strategies have not been fully revealed. Here, we conducted a genome-wide screen of genes important for biofilm regulation using atmospheric and room temperature plasma mutagenesis together with the whole-genome resequencing technology. Three genes (*D7M10_RS02105*, *D7M10_RS27690,* and *D7M10_RS25705*) encoding GGDEF-EAL domain-containing proteins were found to have different mutation manifestations between biofilm cells and free cells. On direct testing, null mutants of *D7M10_RS02105* and especially *D7M10_RS27690* exhibited significantly elevated cyclic di-GMP (c-di-GMP) levels. Further studies indicated that a higher level of c-di-GMP caused by the null mutant of *D7M10_RS27690* triggered cell growth, the production of siderophore and exopolysaccharide as well as autoaggregation, and hindered cell motility, all of which together promote biofilm formation. RNA-sequencing analysis revealed the transcription profile regulated by *D7M10_RS27690*, mostly including flagellar assembly and peptidoglycan biosynthesis pathways. Therein, the downregulated genes enriched in flagellar assembly were verified by qRT-PCR; the result of which was in agreement with the decreased cell motility.

**IMPORTANCE:**

Biofilms formed by spoilage bacterium *Pseudomonas fluorescens* will bring about food quality and safety issues. In this study, we present the establishment of a genetic method and verified its reliability and efficiency for identifying genes associated with biofilm regulation. The genes we discovered offer new perspectives on the mechanisms of biofilm regulation in spoilage bacterium *P. fluorescens*. Moreover, the gene screen method based on atmospheric and room temperature plasma mutagenesis and whole-genome resequencing-coupled technology overcomes the labor-intensive issues caused by traditional methods and should generally be suitable for identifying genes associated with biofilm formation or dispersion in other bacteria.

## INTRODUCTION

Biofilms consist of clusters of microbial cells that adhere to both living and non-living surfaces, where they become encased in a matrix of extracellular polymeric substances (EPS) (1). The composition of the matrix varies significantly among different bacterial species; however, it typically consists of exopolysaccharides, protein-based components such as carbohydrate-binding proteins, adhesins, or amyloid fibers, along with extracellular DNA (2). The matrix endows biofilms with high resistance to external stress, such as antibiotics, and therefore makes the biofilm very difficult to eradicate. In the food industry, biofilms are usually formed on food and food contact surfaces, which will serve as reservoirs for the recurrent contamination and offer substrates for the growth of other pathogens and spoilage bacteria, further threatening the food quality and safety (3).

*Pseudomonas fluorescens* is a gram-negative bacterium, which is broadly present in various environments and able to live within an extensive range of temperatures. Its psychrotrophic nature and high proteolytic, lipolytic, and lecithinase activities make it not only a dominant spoilage organism of dairy products, meat products, and pre-processed fresh vegetables (4–6), but also the specific spoilage organism (SSO) of many precious aquatic products like turbot, tuna, salmon, etc. (7, 8). Due to the fact that biofilm is the primary growth mode of bacteria found in natural environments, uncovering the biofilm regulation mechanisms of *P. fluorescens* is of great significance for biofilm control and food preservation. Bacteria usually employ a range of strategies to develop biofilms, and different strains might use distinctive strategies. Until now, a series of strategies involved in biofilm formation have been reported, with the second messenger cyclic di-GMP (c-di-GMP), cyclic di-AMP, and quorum sensing being the most widely applicable regulatory mechanisms in bacteria (9). Although there have been whole-genome screens focusing on biofilm formation in *P. fluorescens* and several studies have reported the role of c-di-GMP and the corresponding metabolic enzymes in biofilm formation, all these studies were carried out focusing on biocontrol strains of *P. fluorescens*, such as *P. fluorescens* WCS365, SBW25, WH6, and pf0-1 (10–13). The biofilm formation strategies for spoilage strains such as *P. fluorescens* PF08 are still not fully understood.

Typically, constructing a transposon mutant library combined with comparing biofilm formation phenotypes is employed to screen the candidate genes required for biofilm formation (14, 15), which is cumbersome and time-consuming. Atmospheric and room temperature plasma (ARTP) is the latest physical method for random microbial mutagenesis. The generated plasma jet can destroy cell walls and membranes and induce significant breakage of DNA strands, resulting in mutational accumulation. Compared with the traditional mutation techniques, ARTP can induce a variety of DNA damages and elevate the mutation frequency through straightforward procedures, less sequence preference, low cost, high safety, and environmental friendliness (16). Therefore, ARTP is widely used as an efficient tool to obtain mutants with the desired phenotypes. In recent times, advancements in genomics and next-generation sequencing technologies have led to the utilization of whole-genome resequencing (WGR) for the detailed mapping of mutation molecular characteristics and identifying candidate genes on a whole-genome scale (17, 18). Hence, resequencing the whole genome of different growth state cells after ARTP mutagenesis is a promising way to high-throughput screen potential biofilm regulatory genes at the whole-genome level.

In this study, we utilized ARTP to generate an abundant mutant library of SSO *P. fluorescens* PF08. Biofilm cells and free cells were collected, respectively, after the biofilm was formed. WGR technology was further applied to analyze the gene mutation differences between biofilm cells and free cells for identifying the candidate genes associated with biofilm formation. Three genes coding for GGDEF-EAL domain-containing protein were screened out for their potential function in c-di-GMP metabolism. Null mutant strains were constructed to explore whether these three genes possess diguanylate cyclase (DGC) and/or phosphodiesterase (PDE) activity as well as their roles in biofilm formation. The global effects of c-di-GMP in *P. fluorescens* PF08 were further explored by RNA-sequencing technology. This study will enhance our comprehension of the regulation mechanism responsible for biofilm formation and provide a theoretical basis for food quality and safety control.

## MATERIALS AND METHODS

### Bacterial strains, plasmids, and culture conditions

The plasmids and bacterial strains utilized in this research are detailed in Table S1. The *Escherichia coli* strains were grown in Luria-Bertani (LB) broth at a temperature of 37°C, while *Pseudomonas fluorescens* strains were maintained at 28°C. If necessary, the culture mediums were enriched with the following chemicals: 50 µg/mL of 2,6-diaminopimelic acid (DAP), 100 µg/mL of ampicillin, 50 µg/mL of kanamycin, and 50 µg/mL of gentamicin (Gen).

### ARTP mutagenesis

The ARTP mutagenesis machine (ARTP-IIS) from Wuxi TMaxTree Biotechnology Co., Ltd. (Wuxi, China) was utilized to construct a mutant library as reported previously (19). A total of 10 µL of *P. fluorescens* PF08 (about 1.0 × 10^8^ CFU/mL) was evenly spread on the sterile copper slide. These slides were subsequently positioned inside the machine chamber. The distance from the slides to the plasma emitter was set at 2 mm, with an output power of 120 W and a carrier gas flow rate of 10.0 L/min. The exposure duration of mutagenesis ranged from 0 to 30 seconds. Following the mutagenesis process, the treated cells underwent biofilm cultivation.

### Whole-genome resequencing

The whole mutant library cells were grown overnight and then were diluted 1:100 in freshly prepared LB medium and incubated in Petri dishes statically at 28°C for 24 h. After cultivation, the supernatant was collected, washed twice with phosphate buffered saline (PBS), and then subjected to centrifugation to obtain free cells, while the biofilm cells were collected using a cell scraper after washing with PBS. DNA samples extracted from free cells are denoted as FgDNA, and those extracted from biofilm cells are denoted as BgDNA, allowing for comparative analysis of genetic material from different cellular states. For each sample, a minimum of 3 µg of genome DNA was utilized to construct the sequencing library. Following Illumina’s standard protocol for genome DNA library preparation, a paired-end library with an approximate insert size of 450 bp was created. The purified genome DNA was fragmented into smaller pieces of the expected length using Covaris, while T4 DNA polymerase was employed to produce blunt ends. Subsequently, an A base was added to the 3′ end of the blunt phosphorylated DNA fragment, and the adapter was ligated to the end. The specific fragments were then purified via gel electrophoresis and subsequently amplified via PCR. Lastly, the quantified Illumina pair-end library will be utilized for Illumina NovaSeq 6000 sequencing (150 bp * 2, Shanghai BIOZERON Co., Ltd). Burrows-Wheeler aligner (BWA) analysis was used to compare the sequencing results with reference genome sequence *P. fluorescens* strain PF08 chromosome (accession: CP032618.1) obtained from NCBI (https://www.ncbi.nlm.nih.gov/nuccore/CP032618.1) (20). After the PCR-duplication reads were eliminated using SAMtools (21), the sequencing depth and coverage relative to the reference genome were calculated. GATK was employed to detect single nucleotide polymorphism (SNP) and small insertions and deletions (INDELs) (22), structural variation (SV) was determined using BreakDancer (23), and copy number variation (CNV) was detected by CNVnator (24).

### Null mutant construction

Null mutants of the target gene were constructed as previously described (25, 26). Bacterial strains, primers, and plasmids utilized here are listed in Tables S1 and S2. Briefly, *attB1* and *attB2* were flanking the fusion sequences of upstream and downstream regions of the target gene for the subsequent recombination with vector pHGM01. The resultant recombination mixture was then introduced into the donor strain *E. coli* WM3064 and cultured on selective plates containing Gm and DAP. The integration of the recombinant vectors into the *P. fluorescens* PF08 chromosome was achieved through conjugation at a 2:1 ratio of donor to recipient. In the final step, colonies that were susceptible to Gen and resistant to sucrose were screened as deletion mutants using PCR, and this identification was subsequently confirmed by DNA sequencing.

### Growth curve determination

We adjusted the overnight culture to OD_600_ 1.0, then diluted it in fresh LB broth at a ratio of 1:100, and finally added 300 µL of the diluted culture to the Bioscreen microplate. Three biological replicates were set for each strain, OD_600_ of which was measured every 2 h using the automatic microbial growth curve analyzer Bioscreen C and cultured at 28°C for 48 h with shaking at 200 rpm.

### c-di-GMP quantification

The extraction of c-di-GMP was carried out as outlined in previous research (26). Cultures grown overnight were subjected to centrifugation, after which the resulting cell pellets were agitated with 100 µL of extraction solution (composed of methanol, acetonitrile, and 0.1 M formic acid in a ratio of 40:40:20) for every 48 mg of wet cell weight. The mixtures underwent ultrasonic treatment for 30 min and were allowed to incubate for an extra 30 min at −20°C. Insoluble components were eliminated through centrifugation at 4°C. The resulting supernatants were collected to quantify the intracellular c-di-GMP using the c-di-GMP ELISA kit (MlBio, Shanghai, China), adhering to the manufacturer’s guideline.

### Biofilm quantification

The biomass of biofilms generated by each strain was assessed using a microtiter plate assay as outlined in a previous study (27). Target strains grown overnight were diluted 1:100 in freshly prepared LB medium and incubated in 12-well microtiter plates at 28°C for 24 and 48 h, respectively, with 10 biological replicates established for each strain. Biofilms were treated with 0.1% crystal violet for 15 min, followed by dissolution of the stained biofilm using 95% ethanol containing 0.05% Triton X-100. The absorbance was recorded at 590 nm with a microplate reader. The wild-type (WT) strain and LB medium served as a control and blank, respectively.

### Scanning electron microscopy (SEM)

Biofilm morphology was analyzed by SEM (28). Polystyrene coverslips (14 mm in diameter) were incubated with diluted overnight cultures at 28°C for 24 h. After incubation, the coverslips underwent two washes with PBS and were fixed in 2.5% glutaraldehyde overnight at 4°C. They were washed using 0.1 M PBS for 15 min each and treated with 1% osmium tetroxide for 1 to 2 h. Following three more washes with 0.1 M PBS, samples were subjected to a series of ethanol solutions (30%, 50%, 70%, 80%, 90%, and 95%) for 15 min per concentration, followed by two 20 min treatments with 100% ethanol. The samples were then soaked in a 1:1 solution of ethanol and isoamyl acetate for 30 min and subjected to pure isoamyl acetate for 1 h or overnight. Finally, critical point drying was conducted for 5 h, after which the samples were coated and examined using Hitachi SU8010 field emission SEM (Hitachi High-Tech, Tokyo, Japan) with the secondary electron detectors. The working voltage was 3.0 kV, and the distance from the sample to the detector was set at 8.5 mm.

### Siderophore quantification

Siderophore was quantified as detailed previously (29). In brief, all target strains were grown in Minimal Media 9 broth (3 g/L KH_2_PO_4_, 0.5 g/L NaCl, 1 g/L NH_4_Cl, 0.095 g/L MgCl_2_, 0.011 g/L CaCl_2_, 32.24 g/L piperazine-N,N'-bis(2-ethanesulfonic acid [PIPES], 2 g/L glucose, 3 g/L Casamino Acid) overnight at a ratio of 1:100. Then, the bacterial culture was centrifuged, an equal volume of supernatants was added to the chrome azurol S (CAS) solution (0.6 g/L CAS, 0.027 g/L FeCl_3_·6H_2_O, 0.73 g/L hexadecyltrimethylammonium bromide [HDTMA], 0.04 g/L HCl), and the mixture was subjected to absorbance measurement after incubation in the dark for 1 h. The CAS/HDTMA complexes bind strongly with ferric iron and produce a blue color. When siderophore chelates the iron from this dye complex, the color shifts from blue to orange. The OD_630_ for the blank control and experimental group is noted as Ar and A, respectively. Cell number of mutant strain and WT was recorded as Nm and Nw, respectively. Finally, the relative siderophore production (%) per cell of WT was calculated as (Ar − A)/Ar × 100/(Nw/Nw), while that of the mutant strain was (Ar − A)/Ar × 100/(Nm/Nw).

### Congo red assay

A total of 5 µL aliquot of culture grown overnight was inoculated onto an agar plate (1.0% tryptone and 1.5% agar), incorporating the Congo red dye (Sigma) (40 µg/ mL) and Coomassie brilliant blue G-250 (Sigma) (20 µg/ mL), and cultured at 28°C. The colony morphology in the identical agar plate was photographed and compared (30).

### Motility assay

A total of 2 µL of an overnight culture was inoculated into the semi-agar medium incorporating 1.0% tryptone, 0.5% NaCl, and 0.3% agar (31). The diameters of the swimming zone were recorded after 12–24 h culturing at 28°C.

### Autoaggregation assay

Autoaggregation was analyzed as detailed before (31). In brief, overnight cultures were re-suspended in PBS at pH 7.4 to achieve an OD_600_ of 1.0. Two milliliters from each sample was distributed into three separate tubes, and the initial OD_600_ was recorded. Then, the tubes were stored at room temperature without agitation. At specified intervals (2 h, 12 h, 24 h), 200 µL was gently taken from the top of the suspension for OD_600_ measurement. Each strain’s measurement was normalized to the initial OD_600_.

### Cell hydrophobicity assay

Cell hydrophobicity determination was modified on the basis of previous studies (32). Overnight cultures were re-suspended in PBS at pH 7.4 to achieve an OD_600_ of 1.0 (initial OD_600_). A total of 1 mL of ethyl acetate was combined with 3 mL of cell suspension and agitated for 1 min. This mixture was allowed to stand for 15 min to facilitate the phases’ separation. Afterward, 200 µL of the aqueous phase was gently taken for OD_600_ measurement (final OD_600_). The affinity to ethyl acetate of each strain was calculated with the formula % hydrophobicity = 100 * [1 − (final OD_600_/initial OD_600_)].

### RNA sequencing and data assessment

Total RNA of *P. fluorescens* PF08 WT and Δri*DR690* was extracted using Trizol, 2 µg RNA was utilized for the stranded RNA-sequencing library preparation using Ribo-off rRNA Depletion Kit (catalog no. MRZG12324, Illumina) and KCTM Stranded mRNA Library Prep Kit for Illumina (catalog no. DR08402, Wuhan Seqhealth Co., Ltd., China) following the manufacturer’s instruction. Library products within 200–500 base pairs were enriched and quantified, and subsequently were sequenced on the DNBSEQ-T7 sequencer (MGI Tech Co., Ltd., China) utilizing the PE150 model. For each RNA-sequencing sample, three separate libraries were generated. Following the filtration of the raw reads, the clean reads were matched to the genome of *P. fluorescens* PF08 (GenBank accession number CP032618.1). Genes differentially expressed between groups were identified using the edgeR package (version 3.12.1) (33). |log2 fold change| > 0.32 and an adjusted *P*-value <0.05 were used to judge the statistical significance of gene expression differences. Kyoto Encyclopedia of Genes and Genomes (KEGG) enrichment analysis for differentially expressed genes was implemented by KOBAS software (version 2.1.1) with a *P*-value cutoff of 0.05 to judge statistically significant enrichment.

### qRT-PCR

Total RNA was subjected to Turbo DNA-free (Thermo Fisher Scientific, Waltham, MA, USA) to delete genome DNA contamination. cDNA synthesis was performed utilizing random hexamers (GE Healthcare, Stockholm, Sweden) along with SuperScript II reverse transcriptase (Invitrogen, Grand Island, NY, USA). The qRT-PCR was conducted as detailed previously (34). The primers utilized for qRT-PCR were provided in Table S3. The relative transcript level of the genes was normalized against the quantity of 16S rRNA.

### Statistical analysis

All data are presented as mean ± SD. Statistical evaluation was conducted using the Student’s *t*-test. A two-tailed paired *t*-test was applied for comparing two groups. A value of *P* < 0.05 was deemed significant.

## RESULTS

### High-throughput strategy for identifying genes involved in biofilm formation

**Fig 1:**
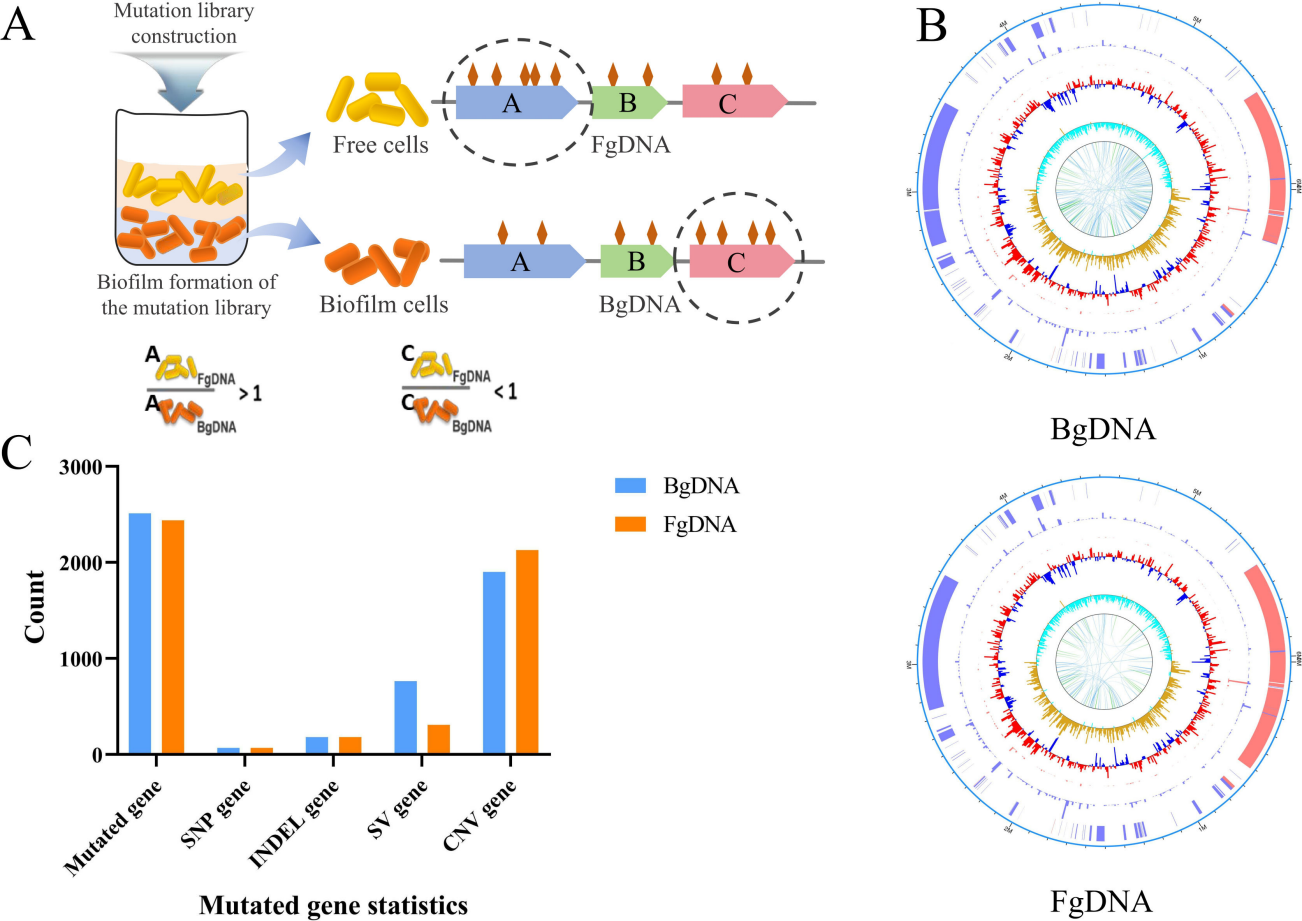
Genome-wide screen for genes involved in biofilm regulation using ARTP mutagenesis combined with WGR technology. (**A**) Schematic diagram of the strategy for identifying genes involved in biofilm regulation. Cells of the mutant library were grown statically in the Petri dish at 28°C and separated into free cells and biofilm cell fractions. DNA was extracted from free cells and biofilm cells, named FgDNA and BgDNA, respectively. After whole-genome resequencing and data analysis, the FgDNA:BgDNA ratio of total mutation counts (including SNP, INDEL, SV, and CNV) for each gene was calculated. As shown in the hypothetical example on the right, gene A and gene C have more and fewer mutations in FgDNA than in BgDNA, and are therefore potentially required for biofilm formation or dispersion. Therefore, we focused on genes in categories A and C. (**B**) The mutation profile of biofilm cells and free cells. The outermost circle represents the genome size. The second circle represents CNV, with red indicating duplication and blue indicating deletion. The third circle represents INDEL, depicted as a standard histogram showing the frequency of insertion/deletion variations in the region. The fourth circle represents SNP, shown as a standard histogram indicating the frequency of SNP variations in the region. The fifth circle represents GC content, with the outward red part indicating GC content higher than the genome-wide average and the inward blue part indicating GC content lower than the genome-wide average. The sixth circle represents the GC skew value, which is positive when the forward strand tends to transcribe CDS (coding sequences) and negative when the reverse strand tends to transcribe CDS. The innermost circle indicates SV links, with forward SVs in blue and reverse SVs in green. (**C**) Statistics of mutant genes in different types of biofilm cells and free cells, including SNP, INDEL, SV, and CNV.

Due to the distinct growth states of free cells and biofilm cells, we can identify genes involved in biofilm formation using genome-wide sequencing of a mutant library. To achieve this, we developed a biofilm utilizing a mixed inoculum of the whole mutant library, the gDNA of biofilm cells (BgDNA) and free cells (FgDNA) were extracted separately, and the frequency of mutation in the genome was compared between the two DNA samples. Based on the principle that loss-of-function mutations are generally more frequent than gain-of-function mutations (35), overrepresentation of mutations in BgDNA would suggest that loss-of-function mutations promote biofilm formation, indicating negative regulatory roles, while underrepresentation would suggest that loss-of-function mutations impair biofilm formation, indicating positive regulatory roles. Among those mutations, SNPs, INDELs, disruptive SVs (e.g., insertions or deletions within coding regions), and deletions in CNVs are more likely to cause loss of function. In contrast, duplications in SVs and CNVs are more likely to enhance gene function by increasing gene dosage. Regardless of the mutation type, we speculated that total mutation counts (including SNPs, INDELs, SVs, and CNVs) in genes associated with biofilm formation would be either underrepresented or overrepresented in BgDNA compared to FgDNA (Fig. 1A).

After the whole-genome resequencing and calculation, the sequencing depth and coverage of BgDNA relative to the reference genome were 960.99 and 93.60%, respectively, while the sequencing depth and coverage of FgDNA were 201.83 and 93.56%. As expected, the mutation profile of biofilm cells was quite different from that of free cells (Fig. 1B). A sum of 2,511 mutant genes were generated in biofilm cells, including 69 genes containing SNP mutations, 180 genes containing INDEL mutations, 763 genes containing SV mutations, and 1,900 genes containing CNV mutations, while a total of 2,438 mutant genes were generated in free cells, including 68 genes containing SNP mutation, 181 genes containing INDEL mutation, 308 genes containing SV mutation, and 2,129 genes containing CNV mutation (Fig. 1C). The specific information of SNP, INDEL, SV, and CNV mutations was exhibited in Table S4. The mutant genes account for 44.8% and 46.2% of the total genome, respectively. Overall, the mutant library constructed by ARTP was diverse and realized a relatively extensive coverage of the entire genome, and the differences between BgDNA and FgDNA offered a basis for high-throughput screening of candidate genes required for biofilm formation.

### Genome-wide identification of genes associated with biofilm formation

**Fig 2:**
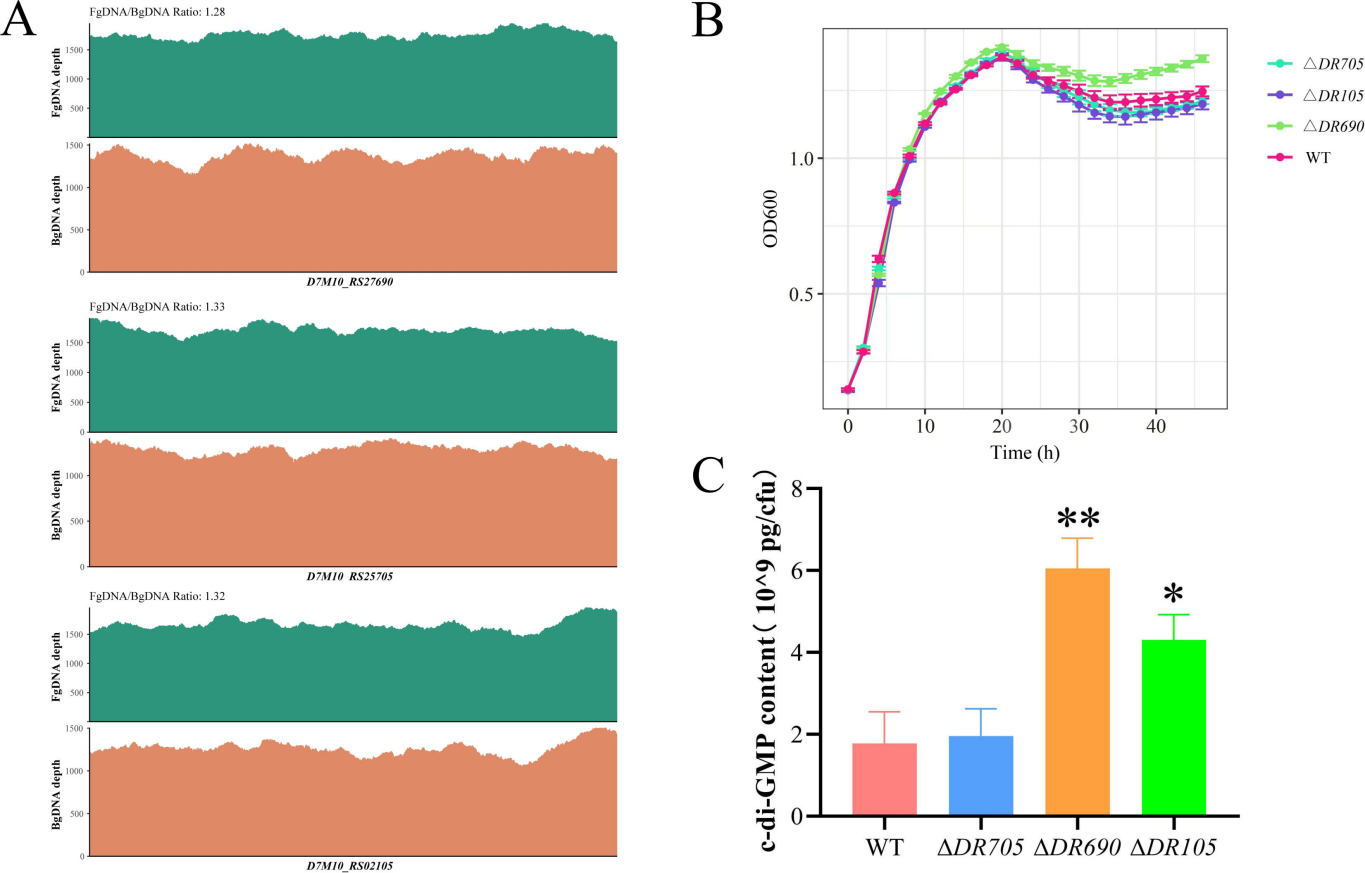
Effects of three EAL domain-containing proteins on c-di-GMP metabolism. (**A**) Sequencing depth spectrum of *D7M10_RS02105*, *D7M10_RS27690,* and *D7M10_RS25705* in FgDNA and BgDNA. (**B**) Growth curves of WT and mutant strains. (**C**) Intracellular c-di-GMP levels of WT and mutant strains. Data were presented as the mean ± standard deviation (*n* = 3, *P < 0.05, **P < 0.01).

Based on the high-throughput screening strategy and principle, we screened out a set of candidate genes closely involved in biofilm formation relying on the KEGG pathways, covering pathways such as quorum sensing, two-component system, biofilm formation, and flagellar assembly (Table S5). It is worth noting that three genes (*D7M10_RS02105*, *D7M10_RS27690,* and *D7M10_RS25705*), which are annotated in NCBI as encoding EAL domain-containing proteins, exhibited different mutation patterns between biofilm cells and free cells, suggesting their potential roles in biofilm formation. The sequencing depth spectrums of these genes are shown in Fig. 2A, where the FgDNA/BgDNA ratio of *D7M10_RS02105* is 1.32, *D7M10_RS27690* is 1.28, and *D7M10_RS25705* is 1.33. Specifically, the CNV of *D7M10_RS02105* is overrepresented in FgDNA relative to BgDNA. Given that CNV deletions are more likely to cause loss of function, we predict that this gene may act as a negative regulator of biofilm formation. In addition, the SVs observed in *D7M10_RS27690* and *D7M10_RS25705* are overrepresented in BgDNA compared to FgDNA, suggesting that they are potentially negative regulators of biofilm formation. However, further experiments are required to validate our predictions.

After domain analysis using SMART (http://smart.embl.de/), these genes were found encoding proteins containing both GGDEF and EAL domains and coupled with signal-sensing domains such as GAF, REC, and PAS (Table 1; Fig. S1A). It is worth noting that both D7M10_RS02105 and D7M10_RS27690 have a degenerate GGDEF domain, which loses its function. In addition, the five most statistically significant motifs were discovered by MEME Suite 5.5.7 (https://meme-suite.org/meme/) and were displayed in Fig. S1B. The function of these conserved motifs was predicted by comparing with known protein databases from the Protein Data Bank using BLASTP 2.16.1+, and results showed that these conserved motifs were highly homologous to the sequence of diguanylate PDE or DGC in *Pseudomonas aeruginosa* (Table S6), which is consistent with the result of our domain analysis. It is well known that the GGDEF domain-containing proteins exhibit DGC activity, synthesizing c-di-GMP, while the EAL domain-containing proteins possess PDE activity, degrading c-di-GMP. This dual regulation of c-di-GMP levels is crucial for controlling various bacterial processes including biofilm formation (26, 36). However, the role of GGDEF-EAL containing proteins in the spoilage strain of *P. fluorescens* (PF08) was undefined.

**TABLE 1:** Domain structure prediction of potential c-di-GMP metabolic proteins of *P. fluorescens* PF08

Protein	GGDEF domain^*a*^	EAL domain	I-site^*b*^
D7M10_RS02105	Rx**ASN**EF	EAL	–
D7M10_RS27690	Rx**S**GDEF	EAL	–
D7M10_RS25705	RxGGDEF	EAL	RxxD

^*a*^ Bold text represents the amino acid residues that have mutations in the GGDEF domain.

^*b*^ ”–” indicates the absence of an I-site in the amino acid sequence.

### Mutants of target genes produce elevated levels of c-di-GMP

We first questioned whether these genes coding for GGDEF-EAL domain-containing protein identified in our screen are equipped with DGC and/or PDE activity. We then constructed null mutants for these genes (named Δ*DR105*, Δ*DR705,* and Δ*DR690*) in the present work.

Before c-di-GMP quantification, we measured the growth curves of each strain because bacterial cells themselves are important constituents of biofilms. As shown in Fig. 2B, the growth level of Δ*DR690* exceeded that of WT after 8 h of culture, the growth gap gradually increased after entering the stationary phase, and the growth of Δ*DR105* and Δ*DR705* was basically consistent with that of WT. Deducting the growth background, the quantity of c-di-GMP per cell produced by Δ*DR690* and Δ*DR105* was approximately 3.4 and 2.4 times that of the WT, respectively, yet Δ*DR705* showed no notable differences with WT (Fig. 2C). These results indicate that these two proteins, D7M10_RS27690 and D7M10_RS02105, generally exhibit PDE activity and can degrade the intracellular c-di-GMP levels, while D7M10_RS25705 makes no contribution to the c-di-GMP pool of *P. fluorescens* PF08.

### Mutants with elevated levels of c-di-GMP formed increased biofilms

**Fig 3:**
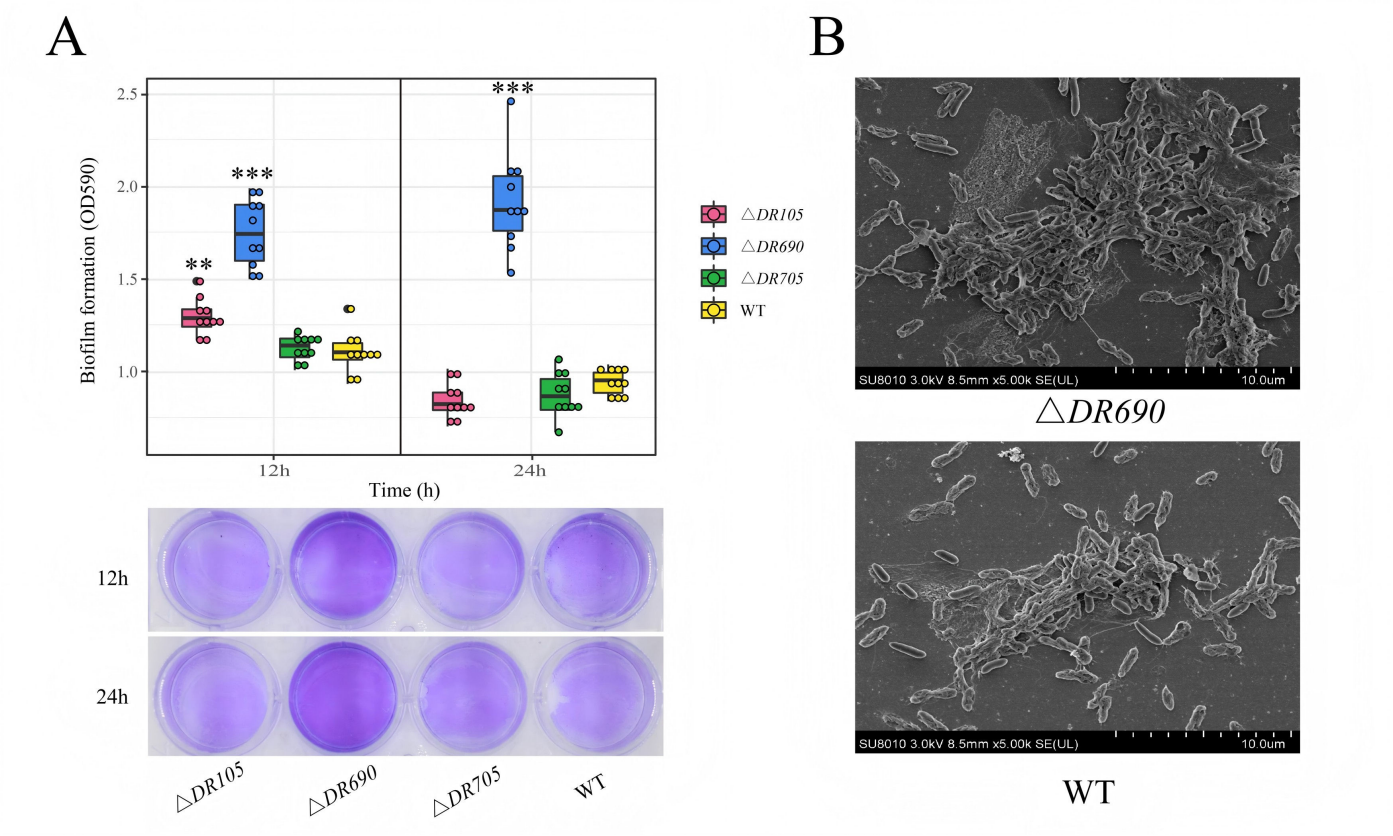
Roles of three EAL domain-containing proteins in biofilm formation. (**A**) Biofilm biomass quantification using the crystal violet method. (**B**) SEM observation of biofilms for WT and Δ*DR690*. Scale bars, 10 µm. Data were presented as the mean ± standard deviation (*n* = 10, **P < 0.01, ***P < 0.001).

We explored the effect of these GGDEF-EAL domain-containing proteins in biofilm formation of *P. fluorescens* PF08 using the crystal violet method. After being cultured for 12 h, the overall biomass of biofilm developed by Δ*DR690* and Δ*DR105* was significantly higher than that of WT, which was 1.6 times (*P* < 0.001) and 1.2 times (*P* < 0.01), respectively, indicating that these mutations enhance biofilm formation compared to the wild type, while Δ*DR705* had no significant difference from that of WT (Fig. 3A). After 24 h of culturing, the overall biofilm biomass of Δ*DR690* was twice that of WT (*P* < 0.001), while the biofilm formation of Δ*DR105* and Δ*DR705* exhibited no notable differences when compared with that of WT (Fig. 3A). The biofilm results of Δ*DR690* and Δ*DR105* were consistent with our prediction, suggesting that *D7M10_RS27690* and *D7M10_RS02105* are negative regulators of biofilm formation. In order to further illustrate the differences in biofilm formation between Δ*DR690* and WT, we further used the SEM to compare the biofilm morphology of Δ*DR690* and WT. Imaging revealed that the biofilm formed by Δ*DR690* contained more extracellular matrix, wrapped more cells, had a higher degree of cellular aggregation, and formed more reticular structures (Fig. 3B). In total, the Δ*DR690* mutant with the highest level of c-di-GMP formed the most abundant biofilms.

### Effects of GGDEF-EAL domain-containing proteins on the siderophore and exopolysaccharide production, cell motility, autoaggregation, and hydrophobicity

In order to clarify how GGDEF-EAL domain-containing proteins regulate the biofilm formation of *P. fluorescens* PF08, we performed a series of behavioral experiments as follows.

**Fig 4:**
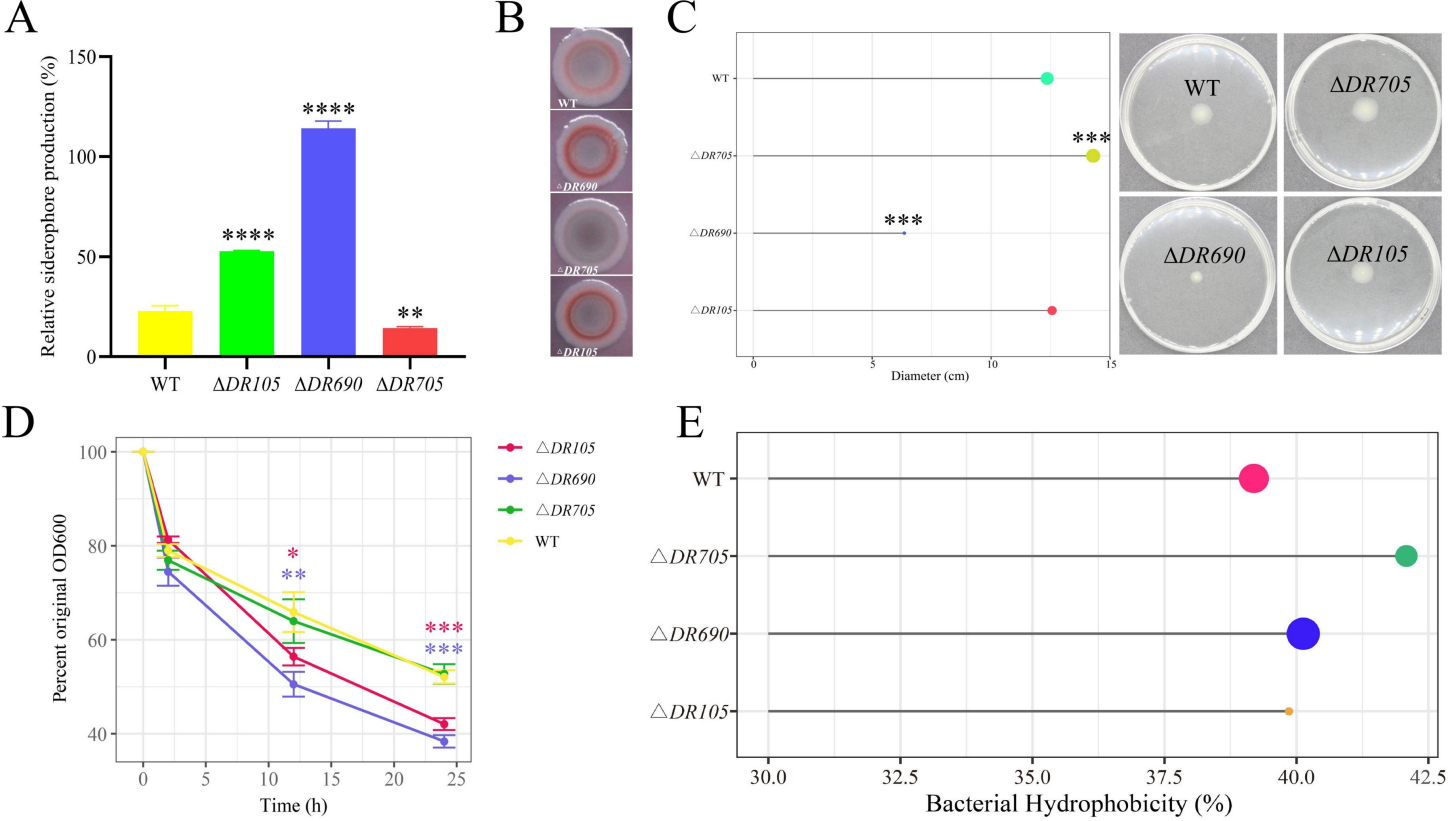
Exploring the biofilm-associated biochemical process regulated by three EAL domain-containing proteins. (**A**) Siderophore relative production of WT and mutant strains. (**B**) Bacterial colony morphology on Congo red agar plates. (**C**) The swimming motility and the swimming zone formed by different strains. (**D**) The cell autoaggregation of WT and mutant strains. (**E**) The cell hydrophobicity of WT and mutant strains. Data were presented as the mean ± standard deviation (*n* = 3, *P < 0.05, **P < 0.01, ***P < 0.001, ****P < 0.0001).

Iron is an essential and rare nutrient for bacteria; its uptake and sequestration are critical for bacterial survival and biofilm formation (37). However, iron mostly exists in the form of poorly soluble ferric iron in the natural environment, which is difficult to utilize (38). *P. fluorescens* PF08 can produce siderophores to overcome the problem of iron bioavailability (29). As depicted in Fig. 4A, when compared to WT, the relative content of siderophore in Δ*DR105* and Δ*DR690* is higher than that in WT, while the relative content of siderophore in Δ*DR705* is lower. The results showed that the deletion of *D7M10_RS02105* and *D7M10_RS27690* can promote the iron-producing capacity of *P. fluorescens* PF08, while deletion of *D7M10_RS25705* will slightly weaken the ability to produce siderophore.

Exopolysaccharide is a vital component of the biofilm matrix (2). Congo red can bind to several exopolysaccharides like cellulose and amyloid fiber and is commonly utilized for the semi-quantitation of exopolysaccharide production (30). By Congo red staining, Δ*DR690* and Δ*DR105* produced redder colonies than those of WT, while Δ*DR705* produced whiter ones (Fig. 4B), indicating that deletion of *D7M10_RS27690* and *D7M10_RS02105* promoted the exopolysaccharide production. The results are in accordance with the previous biofilm biomass results.

Bacterial motility is crucial for biofilm dispersion (2). As shown in Fig. 4C, after 18 h of culturing, the swimming zone formed by WT was 1.9 times that of Δ*DR690* (*P* < 0.001), and that formed by Δ*DR705* was 1.2 times that of WT (*P* < 0.001). Nevertheless, no notable differences were noticed between Δ*DR105* and WT. These results indicate that the deletion of *D7M10_RS27690* largely reduced cell motility, which can account for the promotion of biofilm formation. On the other hand, the deletion of D7M10_RS25705 slightly enhanced cell motility.

Bacteria and their surface elements can interact with each other to promote the aggregation among bacterial individuals, thereby facilitating the formation of biofilms (39). After 12 h of cell standing, the amounts of the upper-layer cells of WT, Δ*DR690*, Δ*DR105*, and Δ*DR705* are 65.87%, 50.54%, 56.40%, and 63.97% of the initial values, respectively (Fig. 4D). After 24 h of standing, they are 52.09%, 38.38%, 42.05%, and 52.70%, respectively (Fig. 4D). Results imply that the deletion of the genes *D7M10_RS27690* and *D7M10_RS02105* can significantly promote cellular autoaggregation.

Bacterial surface hydrophobicity plays a vital role in bacterial adhesion (28). As shown in Fig. 4E, the hydrophobicity of the deleted strains Δ*DR105*, Δ*DR690,* and Δ*DR705* had no significant differences compared to that of WT, indicating that deletions of *D7M10_RS02105*, *D7M10_RS27690,* and *D7M10_RS25705* have no effects on the bacterial surface hydrophobicity.

### Transcriptome analysis revealed biofilm-related genes regulated by protein D7M10_RS27690

**Fig 5:**
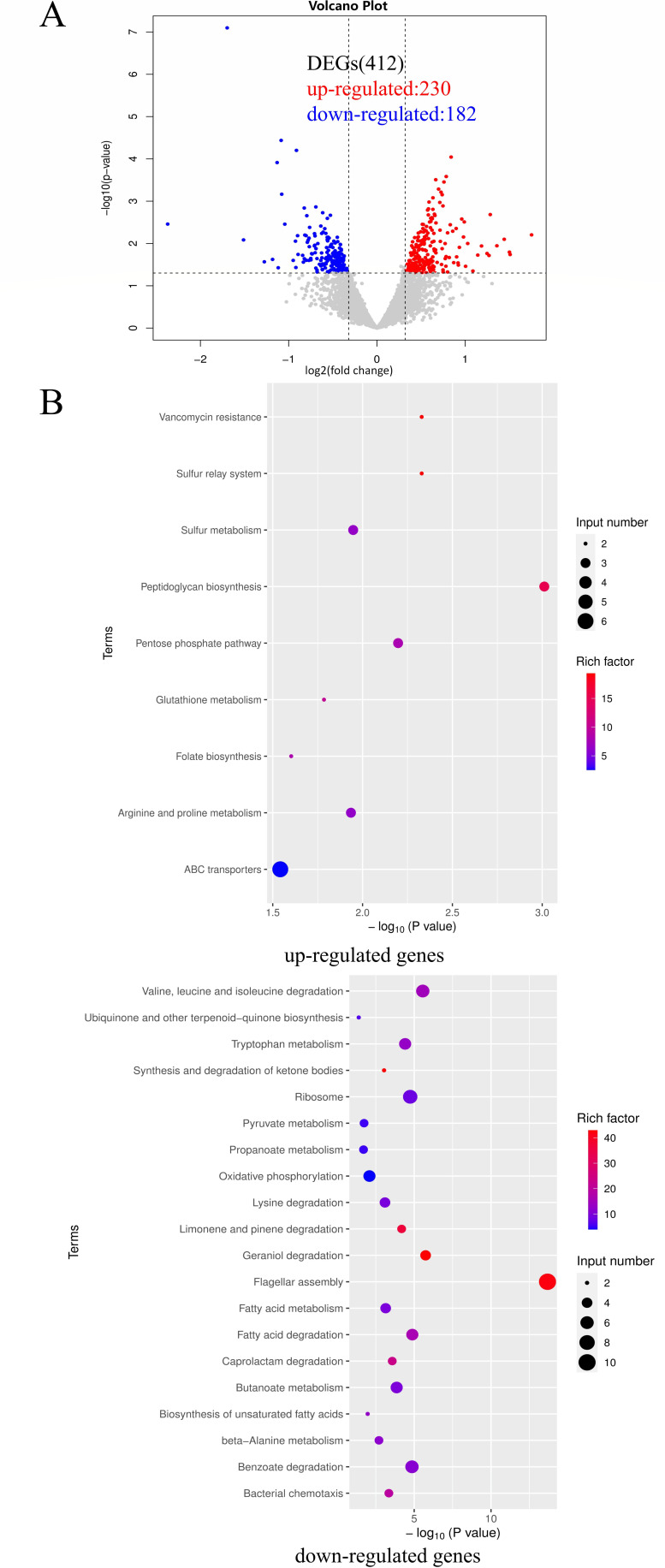
Transcriptomic analysis of Δ*DR690* compared with WT using RNA sequencing. (**A**) The volcano plot of DEGs in Δ*DR690* compared to WT. Red circles are upregulated genes, and blue circles are downregulated genes. (**B**) Scatter diagram of KEGG enrichment pathways for upregulated and downregulated genes of Δ*DR690*, respectively. |log2 fold change| > 0.32 and an adjusted *P*-value <0.05 were used to judge the statistical significance of gene expression differences. KEGG enrichment analysis for DEGs was implemented by KOBAS software (version: 2.1.1) with a *P*-value cutoff of 0.05 to judge statistically significant enrichment.

**Fig 6:**
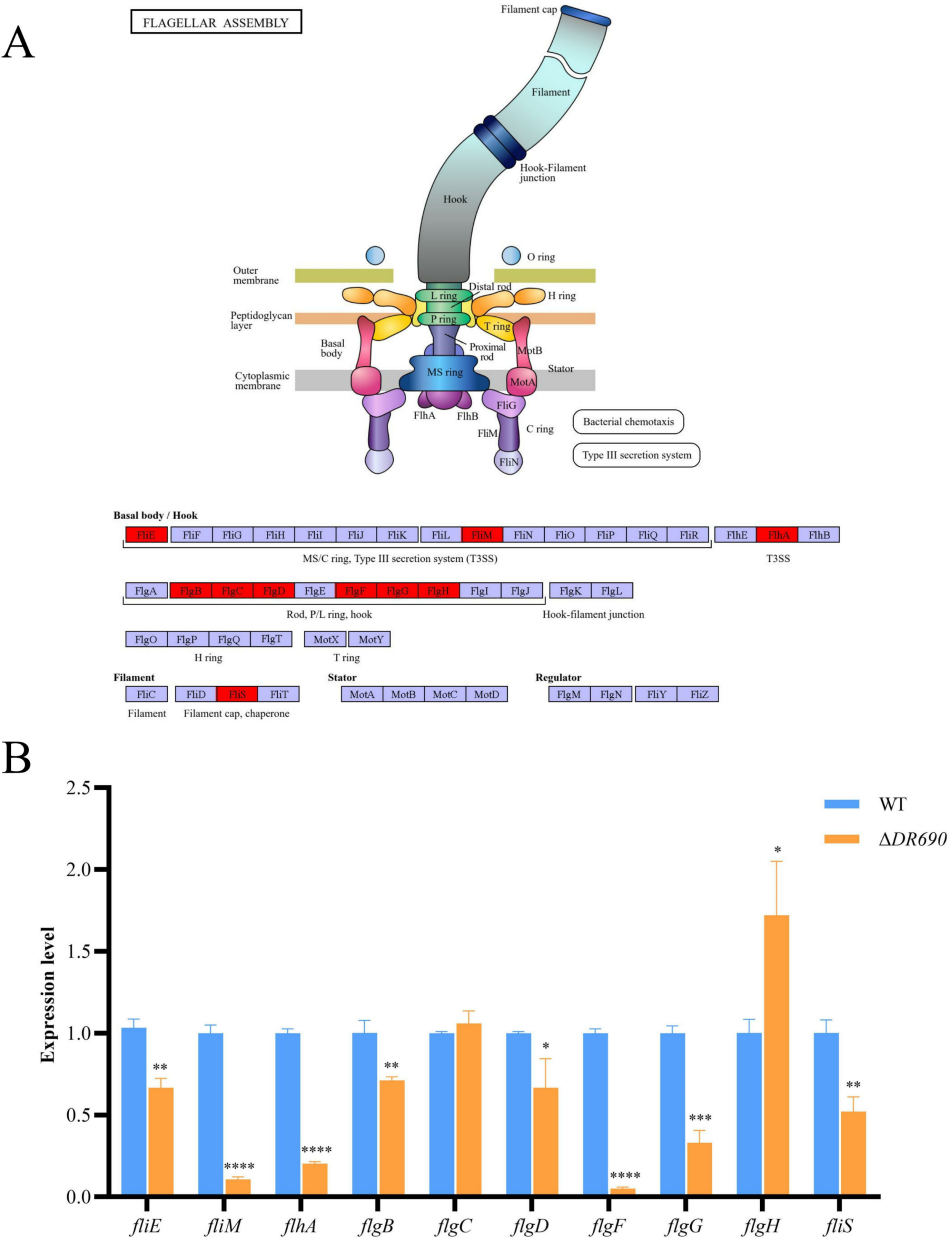
qRT-PCR verification of flagellar assembly-related genes obtained from transcription analysis. (**A**) The positions of enriched genes in the flagellar assembly pathway. (**B**) qRT-PCR verification of the transcription of genes enriched in the flagellar assembly pathway. Data were presented as the mean ± standard deviation (*n* = 3, *P < 0.05, **P < 0.01, ***P < 0.001, ****P < 0.0001).

To explore the regulatory role of protein D7M10_RS27690 in biofilm formation of *P. fluorescens* PF08, transcriptome profiles of WT and Δ*DR690* were assessed using RNA sequencing. A sum of 412 differentially expressed genes (DEGs) was discovered in Δ*DR690* compared to WT, including 230 upregulated genes and 182 downregulated genes (Fig. 5A). KEGG pathway enrichment was thereafter carried out to screen key genes concentrated in biofilm-related pathways. Compared with WT, the first three significantly enriched pathways of upregulated genes of Δ*DR690* were peptidoglycan biosynthesis, sulfur relay system, and vancomycin resistance (Fig. 5B), and that of downregulated genes were flagellar assembly, geraniol degradation, and valine, leucine, and isoleucine degradation (Fig. 5B). Among them, the enrichment of downregulated genes in flagellar assembly is corresponding with the reduced cellular motility of Δ*DR690*, indicating that D7M10_RS27690 regulates biofilm formation mainly through flagellar assembly-dependent cell motility. Specifically, the downregulated genes enriched in the flagellar assembly pathway are *fliE*, *fliM*, *flhA*, *flgB*, *flgC*, *flgD*, *flgF*, *flgG*, *flgH*, *fliS*; their positions in the pathway were exhibited in Fig. 6A. We further verified using qRT-PCR that the transcript level of all these genes except for *flgC* and *flgH* was significantly attenuated in ∆*DR690* (Fig. 6B), which agreed with RNA-sequencing data.

Apart from those KEGG enrichment genes, we found an ABC transporter coding gene *D7M10_RS10480* (*pdvE*), which was proven to be responsible for siderophore production in *P. fluorescens* PF08 in our previous study (29), was positively regulated in ∆*DR690* (Table S7). This finding matches our siderophore quantification results.

## DISCUSSION

The formation of biofilms on food or food contact surfaces is a prerequisite for bacteria to spoil foods. Uncovering the biofilm formation strategy of the SSO *P. fluorescens* PF08 is the key to developing effective prevention and control strategies to reduce food spoilage and ensure food safety. Unlike traditional methods such as transposon mutagenesis, which may allow for more controlled mutagenesis and ensure that only one mutation occurs per chromosome, the ARTP mutagenesis approach employed in this study is non-specific and can result in multiple mutations across the genome within a single bacterial cell, including SNPs, INDELs, SVs, and CNVs (16). This provides a powerful tool for generating a broad and diverse mutant library. However, while this broad mutagenesis introduces complexity, it also enables a comprehensive analysis of genes involved in biofilm formation. To mitigate the challenges posed by multiple mutations, whole-genome resequencing technology was combined to efficiently screen the biofilm-associated genes in the SSO *P. fluorescens* PF08. Furthermore, subsequent null mutations and functional analyses will further confirm the reliability of our screen strategy, ensuring that the identified genes play important roles in biofilm regulation. Consequently, many pathway genes closely related to biofilm formation were discovered (Table S5). Although the functions of these genes have not yet been elucidated in the spoilage bacterium *P. fluorescens* PF08, some of them have been proven important for the biofilm formation of other species or strains of the *Pseudomonas* spp. For example, the *hfq* gene enriched in the KEGG quorum-sensing pathway was reported to exert a beneficial influence on the development of biofilms of the biocontrol bacterium *P. fluorescens* 2P24 (40). The *wspC* gene in the two-component system pathway has been certified to promote the biofilm formation of *Pseudomonas putida* (41). The *pslD* gene in the biofilm formation pathway plays a vital role in the synthesis of exopolysaccharide in *P. aeruginosa*, which constitutes the primary element within the biofilm matrix (42). The discovery of these genes provides a research basis and targets for the subsequent in-depth investigation of the biofilm formation mechanism of *P. fluorescens* PF08.

The most intriguing genes that emerged in our study are *D7M10_RS02105*, *D7M10_RS27690,* and *D7M10_RS25705* encoding GGDEL-EAL domain-containing proteins, which are involved in the c-di-GMP metabolism (36). Typically, elevated levels of c-di-GMP stimulate bacteria to transition from the planktonic lifestyle to the biofilm state, a phenomenon extensively documented in various model pathogens, e.g., *Xanthomonas campestris* pv. campestris (Xcc), *Salmonella enterica*, and *P. aeruginosa* (43–45). C-di-GMP was also reported to encourage the biofilm formation of another spoilage bacterium, *Shewanella baltica* OS155, in our previous study (26). It is well known that c-di-GMP is synthesized by DGCs featuring the GGDEF motif, while its breakdown is carried out through PDEs that possess either the EAL or HD-GYP motif (36). According to the NCBI database, we found that there are a large number of genes annotated as coding for GGDEF and EAL/HD-GYP domain protein in the genome of *P. fluorescens* PF08, including 6 genes encoding GGDEF domain proteins, 15 genes coding for EAL/HD-GYP domain protein, and 3 genes encoding tandem domain protein. However, currently in *P. fluorescens* PF08, there are no DGC and PDE proteins with assigned actual functions, and the biological functions of c-di-GMP have not yet been elucidated. Considering our genome-wide screening results and the existing literature reports, we reasoned that these GGDEF-EAL domain-containing proteins can impact the biofilm formation via regulating the intracellular c-di-GMP level in *P. fluorescens* PF08.

We introduced null mutations in the three genes encoding GGDEF-EAL domain-containing proteins and found that deletions of *D7M10_RS27690* and *D7M10_RS02105* markedly elevated the c-di-GMP level, while the deletion of *D7M10_RS25705* had no obvious effects (Fig. 2C), indicating that D7M10_RS27690 and D7M10_RS02105 primarily exhibit PDE activity responsible for c-di-GMP degradation, which aligns with the presence of their degenerate GGDEF domains. Furthermore, we carried out a series of biochemical phenotype experiments to explore the relation between c-di-GMP and biofilm formation in *P. fluorescens* PF08 (Fig. 2B, 3, and 4). It was found that both *D7M10_RS27690* and *D7M10_RS02105* mutants with increased c-di-GMP level can promote biofilm formation through the increased exopolysaccharide production and/or the decreased cell motility of *P. fluorescens* PF08 to different degrees, which basically match those observed in biocontrol strain of *P. fluorescens* and pathogens. For instance, the PDE mutant Δ*dipA* of *P. fluorescens* SBW25 formed a denser biofilm through diminished swarming ability and increased production of EPS (11). The *Escherichia coli* PDE mutant Δ*yhjH* exhibited elevated c-di-GMP levels and strongly decreased cell motility (46). In *Vibrio cholerae*, a PDE mutant Δ*cdgJ* with elevated levels of c-di-GMP exhibited reduced motility and formed improved biofilms (47). Besides, elevated levels of c-di-GMP stimulated the synthesis of an EPS and dramatically enhanced biofilm formation of *Vibrio vulnificus* (48). In *P. aeruginosa*, DGC proteins WspR and YfiN promoted the exopolysaccharide production and biofilm formation via triggering the production of c-di-GMP (49, 50). Taken together, it is proposed that c-di-GMP can regulate biofilm formation in different species under the control of their own DGCs and PDEs. Interestingly, the deletion of D7M10_RS25705 with the GGDEF domain restrained the siderophore production and improved the cell motility, although it had no obvious impact on c-di-GMP levels. Since D7M10_RS25705 has conserved allosteric inhibition site (I-site) with the RxxD motif in the GGDEF domain (Table 1), the binding of c-di-GMP to the I-site will lead to a reduction in the activity of DGC through allosteric inhibition (51). This finding is similar to the previous study in *P. aeruginosa* that a GGDEF domain protein PelD had no DGC activity but largely influenced exopolysaccharide production. Further investigations demonstrated that PelD acted as a c-di-GMP binding protein at an I-site to regulate the biosynthesis of PEL exopolysaccharide (52). As a result, we speculate that D7M10_RS25705 may regulate cell motility as a receptor by interacting with c-di-GMP, while the exact mechanism requires additional investigation. In addition, autoaggregation can contribute to biofilm formation and is usually promoted by exopolysaccharide and/or bacterial adhesin (28, 39). Here, both Δ*DR690* and Δ*DR105* exhibited enhanced autoaggregation, which can be accounted for by the increased exopolysaccharide production and also explain the increased formation of biofilms.

In the transcriptome analysis represented by Δ*DR690*, we found that downregulated genes were significantly enriched in the flagellar assembly pathway when compared with WT, including genes *fliE*, *fliM*, *flhA*, *flgB*, *flgC*, *flgD*, *flgF*, *flgG*, *flgH*, and *fliS* (Fig. 6A). These genes are essential in the process of bacterial flagellar morphogenesis and are part of the flagellar regulon cascade, a hierarchical network of gene expression that controls the sequential assembly of the flagellar structure. In this cascade, flagellar genes are divided into three classes based on their position in the transcriptional hierarchy. Class 1 genes are responsible for initiating flagellar assembly, primarily by activating the expression of downstream genes. Class 2 genes are involved in the formation of the hook-basal body complex, and class 3 genes are essential for the assembly of the flagellar filament. Any mutations occurring in the class 2 hook-basal body genes not only disrupt the proper formation of hook-basal body structures but also impede the transcription of class 3 operons (53). For example, a *fliS* deletion strain was non-motile and carried severely truncated flagella (54); a *flgD* knockout caused the hindrance to the assembly of flagellar hooks and impairment of cell motility (55); disruption of *flgC* resulted in a lack of filament assembly and a reduction in cell motility (56, 57). Based on the above, our qRT-PCR results verified that elevated levels of c-di-GMP could inhibit cell motility through interfering with the expression of *fliE*, *fliM*, *flhA*, *flgB*, *flgD*, *flgF*, *flgG*, and *fliS* of *P. fluorescens* PF08 (Fig. 6B). It is noteworthy that upregulated genes in Δ*DR690* were most significantly enriched in the peptidoglycan biosynthesis pathway, including *murD*, *ddl*, and *mraY*. These genes are involved in the early steps of peptidoglycan biosynthesis. Among them, *murD* encodes D-glutamic acid-adding enzyme that catalyzes the addition of D-glutamic acid to UDP-N-acetylmuramoyl-L-alanine, a crucial cytoplasmic step in the process of synthesizing bacterial cell wall peptidoglycan (58); *ddl* encodes D-Ala-D-Ala ligase, which plays a crucial role in the formation of the dipeptide D-Ala-D-Ala, a vital precursor in the synthesis of bacterial peptidoglycan (59); and *mraY* encodes phospho-MurNAc-pentapeptide translocase, an enzyme that catalyzes the initial step of the lipid-linked cycle in bacterial peptidoglycan biosynthesis (60). Although these genes are not directly targeted at biofilm formation, recently, it has been reported that interfering with peptidoglycan synthesis triggered the dissociation of amyloid-like fibers which linked to the peptidoglycan and reduced the content of exopolysaccharides and proteins in the EPS, compromised the structural integrity of EPS, and led to the disintegration of the biofilm (61). Along with our result, it is possible that c-di-GMP regulates biofilm formation of *P. fluorescens* PF08 through interfering with peptidoglycan biosynthesis.

The importance of siderophore biosynthesis in cell motility as well as biofilm formation has been established in several pathogens. In *P. aeruginosa* PAO1, deletion of genes *pvdI* and *pvdL* required for the siderophore synthesis substantially impaired the biofilm biomass, thickness, and structure (62). In *Staphylococcus epidermidis*, the deletion of a *sfaABCD* gene cluster that is responsible for siderophore biosynthesis was shown to negatively impact biofilm development (63). Strain of *P. putida* lacked in the siderophore biosynthetic gene *ppsD* and had defects in cell motility (64). Mutations in *iucA* and *iutA* responsible for siderophore production resulted in a decrease in cell motility of *Pantoea stewartii* (65). Surprisingly, Δ*DR690* and Δ*DR105* mutated in c-di-GMP degradation were revealed to produce increased siderophores than WT in this study (Fig. 4A), which first reveals the positive association between c-di-GMP and siderophore production. Actually, our previous study discovered that the deletion of the *pvdA* and *pdvE* involved in siderophore biosynthesis significantly enhanced motility, attenuated exopolysaccharide production, and reduced biofilm formation in *P. fluorescens* PF08 (29), indicating the important role of *pvdA* and *pdvE* in biofilm formation. According to our transcriptome results, *pdvE* was significantly upregulated in ∆*DR690* (Table S7). Combined with our phenotype data, it is therefore suggested that c-di-GMP can directly regulate the extracellular polysaccharides production, cell motility, and biofilm formation of *P. fluorescens* PF08 or indirectly via *pvdE*-dependent siderophore production.

Ultimately, we have discovered previously unrecognized genes that play an essential role in the regulation of c-di-GMP level and biofilm formation in SSO *P. fluorescens* PF08. Our results suggest that increased levels of c-di-GMP promote biofilm formation by inducing the production of siderophores and exopolysaccharides, while simultaneously inhibiting cell motility that depends on flagellar assembly. The identified genes in this study offer potential targets for future treatments aimed at inhibiting biofilm formation and preventing food spoilage associated with *P. fluorescens* PF08. Also, our strategy of screening genes in which mutations are underrepresented or underrepresented in BgDNA compared to FgDNA could be applicable to identifying genes responsible for biofilm formation of other microorganisms.

## Data Availability

The RNA-sequencing data are available in Figshare at https://doi.org/10.6084/m9.figshare.28675076.v1. The whole-genome resequencing data are available in the Sequence Read Archive (SRA) under the accession number PRJNA1241504.

## SUPPLEMENTAL MATERIAL

Supplemental material aem.00218-25-s0001.docx Tables S1 to S3, S5, and S6; Figure S1.

Table S4 aem.00218-25-s0002.xlsx All SNPs, INDELs, SVs, and CNVs detected in this study.

Table S7 aem.00218-25-s0003.xlsx Differentially expressed genes between Δ*DR690* and WT.

ASM does not own the copyrights to Supplemental Material that may be linked to, or accessed through, an article. The authors have granted ASM a non-exclusive, world-wide license to publish the Supplemental Material files. Please contact the corresponding author directly for reuse.
