# Supplementary material for: ARTP mutagenesis for genome-wide identification of genes important for biofilm regulation in spoilage bacterium Pseudomonas fluorescens PF08
Source: Appl Environ Microbiol. 2025 May 7;91(6):e00218-25. doi: 10.1128/aem.00218-25 (PMC12175535; doi:10.1128/aem.00218-25)
Supplement: Supplemental material — Tables S1 to S3, S5, and S6; Figure S1. [file aem.00218-25-s0001.docx]

| Table S1 Bacterial strains and plasmids used in this study | | |
| --- | --- | --- |
| **Strains or plasmids** | **Description** | **Reference or source** |
| ***E. coli* strains** |  |  |
| *E. coli* DB3.1λ | Host for pHGM01 | Addgene, Watertown, MA, USA |
| *E. coli* WM3064 | Host for *pir*-dependent plasmids and donor strain for conjugation; RP4 (*tra*) in chromosome; Δ*dapA* | BioVector NTCC, Pekin, China |
|  |  |  |
| ***P. fluorescens* strains** | | |
| *P. fluorescens* PF08 | Wild-type | Lab storage |
| Δ*DR105* | *P. fluorescens* PF08 mutant strain with *D7M10_RS02105* gene deletion | This study |
| Δ*DR690* | *P. fluorescens* PF08 mutant strain with *D7M10_RS27690* gene deletion | This study |
| Δ*DR705* | *P. fluorescens* PF08 mutant strain with *D7M10_RS25705* gene deletion | This study |
|  |  |  |
| **Plasmids** | | |
| pHGM01 | ApR, GmR, CmR; *mob*+ *att*-based suicide vector | Lab storage |

Table S2 Target genes and primers used for mutant construction

| Gene | Primer name | Primer Sequences（5’-3’） |
| --- | --- | --- |
| *D7M10_RS02105* | up-F | GGGGACAAGTTTGTACAAAAAAGCAGGCTACTGGCTTCACGCCAATCG |
|  | up-R | GGTCCGGGTTCGCTATCTATTAATTGCGTCACGACCTCGG |
|  | down-F | ATAGATAGCGAACCCGGACCCAGGCTCAAACGCTATCCGC |
|  | down-R | GGGGACCACTTTGTACAAGAAAGCTGGGTTACTGGCTGCCAATGTCATTGCC |
| *D7M10_RS27690* | up-F | GGGGACAAGTTTGTACAAAAAAGCAGGCTATGCCCATCAACTGCTCAATGCG |
|  | up-R | GGTCCGGGTTCGCTATCTATAATGCTTGCGGACAACCTGCTC |
|  | down-F | ATAGATAGCGAACCCGGACCGAAGGACTGGCGGATTTGTTACG |
|  | down-R | GGGGACCACTTTGTACAAGAAAGCTGGGTTTCCATCAGGGTGTGCAGGGTG |
| *D7M10_RS25705* | up-F | GGGGACAAGTTTGTACAAAAAAGCAGGCTATCTGACCGATCACGATCTTGCC |
|  | up-R | GGTCCGGGTTCGCTATCTATGGAGCGCATTCCATGCTGCATC |
|  | down-F | ATAGATAGCGAACCCGGACCATATTTCCCAGTGGCCGCAG |
|  | down-R | GGGGACCACTTTGTACAAGAAAGCTGGGTTCCATGCTCGCTTCCTCTTGG |

Table S3 Flagella-related genes and primers used for qRT-PCR

| **Gene** | **Gene name** | **Primer sequence** |
| --- | --- | --- |
| D7M10_RS08075 | *flgF* | F: GAAGACGGCACCATCAGCATCC  R: CGGCTTGACCAGCTTGATACGG |
| D7M10_RS08080 | *flgG* | F: CAGACCGCCGACTTCATCAACC  R: GGAGGTTTCCAGGGTGCTTTGC |
| D7M10_RS08085 | *flgH* | F: CGACATCATCACCATCACCCTCAAC  R: TTCAAGCCACCGCCAAACAGC |
| D7M10_RS08130 | *fliS* | F: CCTATGAGAGCCCTTCGCCAATAC  R: GCCGCCTTCCATCAGCATCTG |
| D7M10_RS08155 | *fliE* | F: AGCCGAAATCCGTCGCACAAG  R: CCTGCTGGGTATCGCTGACTTTG |
| D7M10_RS08215 | *fliM* | F: GGCGGTGTGCAGGTGATGAAG  R: GGCTTGATCTTGACCAGGTTGAGG |
| D7M10_RS08255 | *flhA* | F: TCGCTGTCGCAACTGCTCAAG  R: TGACTCTTGGCGGCATTGTTGG |
| D7M10_RS20370 | *flgD* | F: CGACAGCGGGTTCTGGTTCTTC  R: CTCCAAGGTCAAGGATGCCAAGTC |
| D7M10_RS20375 | *flgC* | F: GGCAGCGACTCGTTGTTCCAG  R: CAGCGGGATGATTGGGTTCGTAG |
| D7M10_RS20380 | *flgB* | F: GACAAGACCAAGAACGGCTCCTTC  R: GCGTGCGATACATCAGCGACTC |
| D7M10_RS00575 | *16s rRNA* | F: GAAGGAACACCAGTGGCGAAGG  R: CATCGTTTACGGCGTGGACTACC |

Table S5 Screening of candidate genes associated with biofilm formation

| **Gene ID** | **Gene name** | **NCBI Gene Description** | **Mutant type** | **Ratio** |
| --- | --- | --- | --- | --- |
| **Quorum sensing** |  |  |  |  |
| D7M10_RS01480 | *secB* | protein-export chaperone SecB | CNV | >1 |
| D7M10_RS02400 | *hfq* | RNA-binding protein Hfq | CNV | >1 |
| D7M10_RS02565 | *livK* | branched-chain amino acid ABC transporter substrate-binding protein | CNV | >1 |
| D7M10_RS02575 | *livH* | branched-chain amino acid ABC transporter permease | CNV | >1 |
| D7M10_RS02580 | *livM* | high-affinity branched-chain amino acid ABC transporter permease LivM | CNV | >1 |
| D7M10_RS02585 | *livG* | ATP-binding cassette domain-containing protein | CNV | >1 |
| D7M10_RS02590 | *livF* | ABC transporter ATP-binding protein | CNV | >1 |
| D7M10_RS26120 | *ftsY* | signal recognition particle-docking protein FtsY | SV | <1 |
| D7M10_RS00370 | *zur* | transcriptional repressor | SV | <1 |
| **Two-component system** |  |  |  |  |
| D7M10_RS05525 | *wspC* | chemotaxis protein CheR | SV | <1 |
| D7M10_RS08315 | *cheW* | chemotaxis protein CheW | SV | <1 |
| D7M10_RS01680 | *mcp* | methyl-accepting chemotaxis protein | CNV | >1 |
| D7M10_RS01685 | *mcp* | methyl-accepting chemotaxis protein | CNV | >1 |
| D7M10_RS02040 | *mcp* | methyl-accepting chemotaxis protein | CNV | >1 |
| D7M10_RS23505 | *mcp* | methyl-accepting chemotaxis protein | SV | <1 |
| D7M10_RS02430 | *mcp* | methyl-accepting chemotaxis protein | CNV | >1 |
| D7M10_RS12640 | *fecI* | sigma-70 family RNA polymerase sigma factor | CNV | <1 |
| D7M10_RS12645 | *fecR* | DUF4880 domain-containing protein | CNV | <1 |
| D7M10_RS14210 | *mdtB* | MdtB/MuxB family multidrug efflux RND transporter permease subunit | SV | <1 |
| D7M10_RS01500 | *ntrC* | nitrogen regulation protein NR(I) | CNV | >1 |
| D7M10_RS01505 | *ntrB* | nitrogen regulation protein NR(II) | CNV | >1 |
| D7M10_RS22005 | *aauR* | sigma-54-dependent Fis family transcriptional regulator | SV | <1 |
| D7M10_RS25060 | *uvrY* | DNA-binding response regulator | SV | <1 |
| D7M10_RS25370 | *fecI* | RNA polymerase sigma factor | SV | <1 |
| D7M10_RS25955 | *chpA* | response regulator | SV | <1 |
| D7M10_RS25960 | *pilJ* | chemotaxis protein | SV | <1 |
| D7M10_RS27405 | *phoR* | phosphate regulon sensor histidine kinase PhoR | SV | <1 |
| **Biofilm formation** |  |  |  |  |
| D7M10_RS10305 | *pslD* | polysaccharide export protein | SV | <1 |
| D7M10_RS10310 | *rfbN* | glycosyltransferase | SV | <1 |
| D7M10_RS00140 | *gcvA* | LysR family transcriptional regulator | CNV | <1 |
| D7M10_RS01535 | *glgP* | glycogen/starch/alpha-glucan phosphorylase | CNV | >1 |
| D7M10_RS19375 | *wecB* | UDP-N-acetylglucosamine 2-epimerase (non-hydrolyzing) | INDEL | >1 |
| D7M10_RS02265 | *cpdA* | 3',5'-cyclic-AMP phosphodiesterase | CNV | >1 |
| D7M10_RS25060 | *gacA* | DNA-binding response regulator | SV | <1 |
| D7M10_RS02550 | *gcvA* | LysR family transcriptional regulator | CNV | >1 |
| D7M10_RS26540 | *gspE* | type II/IV secretion system protein | SV | <1 |
| D7M10_RS02860 | *retS* | hybrid sensor histidine kinase/response regulator | SV | >1 |
| D7M10_RS04955 | *gcvA* | LysR family transcriptional regulator | SV | >1 |
| **Flagellar assembly** |  |  |  |  |
| D7M10_RS08115 | *fliC* | flagellin | SNP/INDEL | >1 |
| D7M10_RS08125 | *fliD* | flagellar cap protein FliD | CNV | <1 |
| D7M10_RS02340 | *motA* | flagellar motor stator protein MotA | CNV | >1 |
| D7M10_RS02345 | *motB* | motility protein MotB | CNV | >1 |
| **EAL domain protein** |  |  |  |  |
| D7M10_RS02105 | *NA* | EAL domain-containing protein | CNV | >1 |
| D7M10_RS25705 | *pleD* | EAL domain-containing protein | SV | <1 |
| D7M10_RS27690 | *NA* | EAL domain-containing protein | SV | <1 |

Table S6 Comparison of the conserved motifs with known protein databases from the Protein Data Bank (PDB)

| **Motif** | **Description** | **Query Cover** | **E value** | **Per. Ident** | **Accession** |
| --- | --- | --- | --- | --- | --- |
| 1 | Chain A, MucR Phosphodiesterase [Pseudomonas aeruginosa PAO1] | 100% | 2e-16 | 64.00% | 5M1T_A |
| 2 | Chain A, Diguanylate cyclase [Pseudomonas aeruginosa] | 98% | 3e-17 | 69.39% | 5M3C_A |
| 3 | Chain A, Diguanylate cyclase [Pseudomonas aeruginosa PAO1] | 88% | 1e-04 | 85.71% | 4ZMU_A |
| 4 | Chain A, Diguanylate phosphodiesterase [Pseudomonas aeruginosa] | 96% | 3e-05 | 65.38% | [5MFU_A](https://www.ncbi.nlm.nih.gov/protein/5MFU_A?report=genbank&log$=prottop&blast_rank=9&RID=TCFK3M90016) |
| 5 | Chain A, Diguanylate cyclase [Pseudomonas aeruginosa] | 100% | 2e-07 | 45.00% | [5M3C_A](https://www.ncbi.nlm.nih.gov/protein/5M3C_A?report=genbank&log$=prottop&blast_rank=3&RID=TCFPEYHR016) |


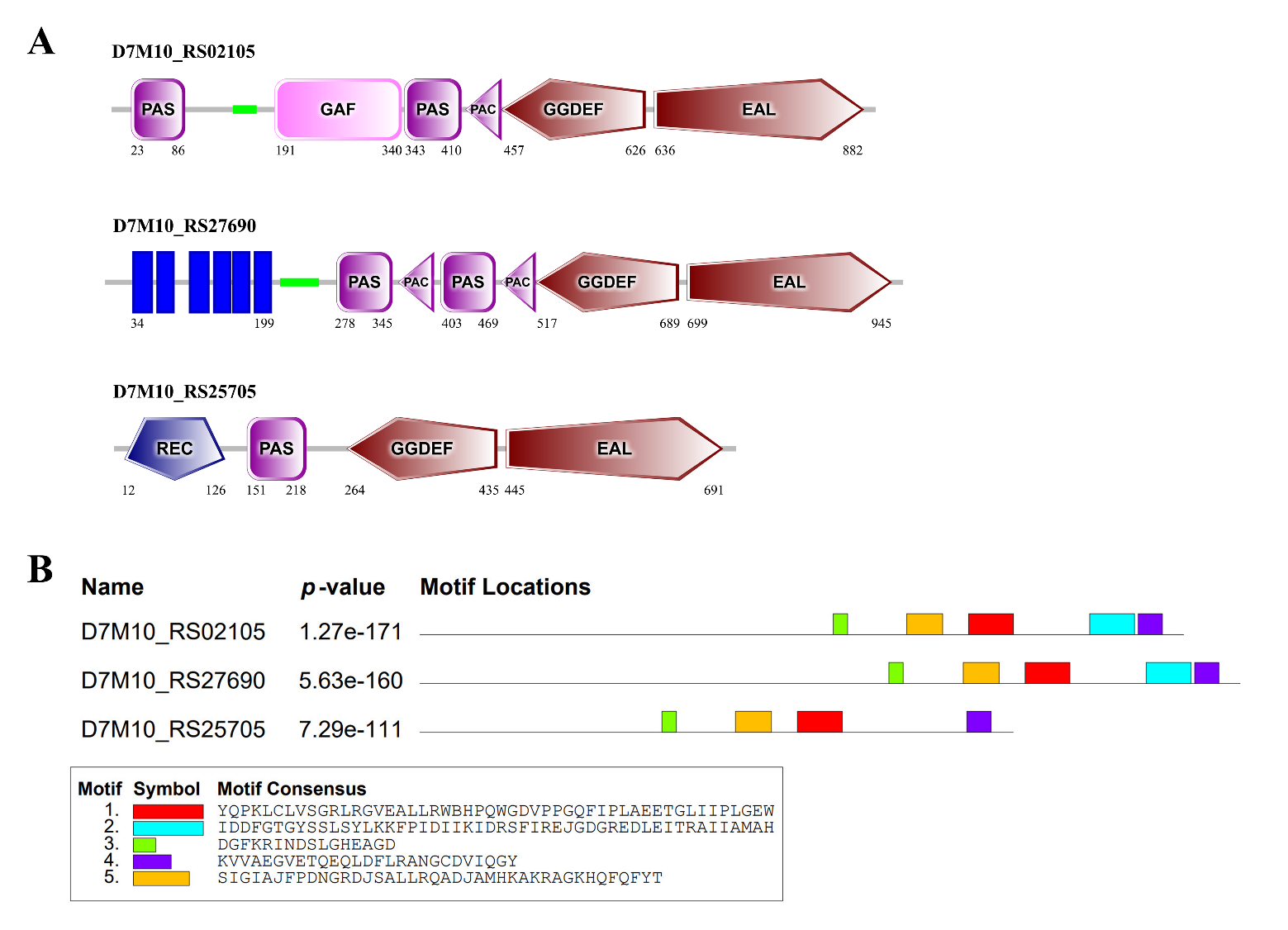


**Fig. S1** (A) Domain analysis of target proteins using SMART. (B) The most statistically significant motifs of target proteins discovered by MEME Suite 5.5.7.
